# Neuronal-specific septin-3 binds Atg8/LC3B, accumulates and localizes to autophagosomes during induced autophagy

**DOI:** 10.1007/s00018-022-04488-8

**Published:** 2022-08-06

**Authors:** Vilmos Tóth, Henrietta Vadászi, Lilla Ravasz, Dániel Mittli, Dominik Mátyás, Tamás Molnár, András Micsonai, Tamás Szaniszló, Péter Lőrincz, Réka Á. Kovács, Tünde Juhász, Tamás Beke-Somfai, Gábor Juhász, Balázs András Györffy, Katalin A. Kékesi, József Kardos

**Affiliations:** 1 ELTE NAP Neuroimmunology Research Group, Department of Biochemistry, Institute of Biology, ELTE Eötvös Loránd University, Budapest, Hungary; 2 Department of Biochemistry, Institute of Biology, ELTE Eötvös Loránd University, Budapest, Hungary; 3 Department of Anatomy, Cell and Developmental Biology, Eötvös Loránd University, Budapest, Hungary; 4 Institute of Materials and Environmental Chemistry, Research Centre for Natural Sciences, Budapest, Hungary; 5 CRU Hungary Ltd., Göd, Hungary; 6 Department of Neuroscience, University of Copenhagen, Copenhagen, Denmark; 7 Department of Physiology and Neurobiology, Eötvös Loránd University, Budapest, Hungary; 8 Laboratory of Proteomics, Institute of Biology, ELTE Eötvös Loránd University, Budapest, Hungary

**Keywords:** Septin, LIR, Atg8, Autophagy, Synaptic pruning, Synaptic autophagy, Neuronal autophagy

## Abstract

In synapses that show signs of local apoptosis and mitochondrial stress and undergo neuro-immunological synapse pruning, an increase in the levels of the presynaptic protein, neuronal-specific septin-3 can be observed. Septin-3 is a member of the septin GTPase family with the ability to form multimers and contribute to the cytoskeleton. However, the function of septin-3 remains elusive. Here, we provide evidence that septin-3 is capable of binding the most-studied autophagy protein Atg8 homolog microtubule-associated protein 1 light chain 3B (LC3B), besides another homolog, GABA receptor-associated protein-like 2 (GABARAPL2). Moreover, we demonstrate that colocalization of septin-3 and LC3B increases upon chemical autophagy induction in primary neuronal cells. Septin-3 is accumulated in primary neurons upon autophagy enhancement or blockade, similar to autophagy proteins. Using electron microscopy, we also show that septin-3 localizes to LC3B positive membranes and can be found at mitochondria. However, colocalization results of septin-3 and the early mitophagy marker PTEN-induced kinase 1 (PINK1) do not support that binding of septin-3 to mitochondria is mitophagy related. We conclude that septin-3 correlates with synaptic/neuronal autophagy, binds Atg8 and localizes to autophagic membranes that can be enhanced with chemical autophagy induction. Based on our results, elevated septin-3 levels might indicate enhanced or impeded autophagy in neurons.

**Supplementary Information:**

The online version contains supplementary material available at 10.1007/s00018-022-04488-8.

## Introduction

Septin proteins can be classified into four homology subgroups, named after a specific member of the group (groups SEPT2, 3, 6, and 7). Most septins bear GTPase function (except for SEPT6 group, in which, due to a substitution, the GTPase function is lost [1]), and can be classified as P-loop NTPases. These proteins can form homo- or heterooligomeric complexes (plausible interactions are between groups 2–6, 3–6, 3–7, and 6–7 [2]) and contribute to the cytoskeleton as filaments, bundles, or rings [3].

In neurons, septins have been associated mainly with morphology, membrane dynamics and transport functions, that conclude to neuronal physiological processes, like the following: (i) Septin-2 plays role in neurite outgrowth [4]. (ii) Septin-11 promotes arborization, dendritic spine generation, and GABAergic synapse connectivity [5]. (iii) Septin-4 and 14 play a role in neuronal migration [6]. (iv) Septin-7 destabilizes microtubules and promotes dendrito- and axonogenesis [7, 8], and also defines polarity [9]. (v) Septin-6 triggers branching along with septin-7, colocalizing with septin-2 and 4 at spine-necks [10–12]. (vi) Septin-5, 4, 8, and 2 are suggested to participate in exocytosis and neurotransmitter release modulation, e.g., by SNARE protein binding [13–17]. (vii) Septin 6 and 7 coordinate multivesicular body biogenesis [18]. (viii) Septins also play a role in store-dependent Ca^2+^ entry to the endoplasmic reticulum (ER) [19, 20].

Neuronal-specific septin-3 (SEPT3) of the SEPT3 subgroup is expressed in the brain and testis with isoforms SEPT3A and SEPT3B, while isoform SEPT3C is not expressed in the brain [21, 22]. It is a soluble or membrane-bound presynaptic protein, but it can be detected in synaptosome-depleted fractions also [23]. To our knowledge, two attempts were made to investigate the role of septin-3 by creating knock-out mice [24, 25], and the absence of septin-3 did not affect neuronal development, viability, or fertility. Additionally, no difference was found in the neuronal growth rate and polarity, synaptogenesis, synaptic transmission, and short-term plasticity of *Septin3*-/- neurons [25]. This led to the conclusion, that septin-3 is negligible for neuronal development and synapse function. However, examining human *postmortem* Alzheimer’s disease (AD) brain samples, Takehashi et al. identified the presence of a polymorphism in *SEPTIN3* (either homo- or heterozygous) as a genetic risk factor for AD [26]. A high-throughput study found a twofold increase of septin-3 protein level in *postmortem* temporal cortical samples of AD patients compared to controls [27]. Septin-3 levels correlate with neuronal development in rat and mouse [21, 28]; also, septin-3 expression is upregulated in retinoic acid-induced neuronal differentiation [22]. Septin-3 levels rise in the visual cortex due to monocular deprivation during development and due aging after an enhanced plasticity period [29].

In our previous study, we showed that septin-3 protein levels positively correlate with the level of C1q tagging of synapses in AD mouse model, APP/PS1 animals [30]. The complement protein C1q associates to dysfunctional synapses that show signs of mitochondrial stress and apoptosis (especially cleaved caspase-3 accumulation) and needed to be cleared by the microglia in a phagoptosis-like elimination [31]. Moreover, by microscopy imaging, we have found positive correlation between the extent of C1q and septin-3 colocalization and septin-3 signal intensity both in wild-type and APP/PS1 animals [30]. In summary, septin-3 accumulates in synapses that are prone to complement-mediated pruning. In light of these correlations, the exact role of synaptic septin-3 needs to be addressed.

Altogether, some earlier studies together with our recent results propose an important role for septin-3, yet the only biological processes associated with septin-3 presently are cellular protein localization, membrane fission and cytoskeleton-dependent cytokinesis (based on GO enrichment analysis [32]). Beyond the better understanding of its basic cellular functions, targeted investigation of septin-3 holds importance in light of its repeatedly described involvement in AD-related molecular alterations.

To find possible binding partners and functional motifs, we investigated septin-3’s sequence for linear motifs, and found Atg8 interactive motifs (AIMs), that are responsible for binding autophagosome forming and related proteins, Atg8s, which in mammals are comprised of two subfamilies: GABARAP(L)s (γ-aminobutyric acid receptor-associated protein (GABARAP), and GABARAP-like proteins 1 and 2) and LC3s (microtubule-associated protein 1 light chain 3 A, B and C). This motif is characteristic of selective autophagy receptors (SARs), which drive specific cargo into the forming autophagophore by binding cargo and Atg8 simultaneously, but can be found in other adaptor proteins related to autophagy, such as those involved in autophagosome transport [33].

A pulldown experiment using septin-3 as bait already identified GABARAPL2 as a binding partner of septin-3 [34].

The object of this study is to examine whether septin-3 is bound to well-known autophagy marker LC3B in vitro and if this interaction is observable in cultured neurons. In addition, we also compared this interaction with SEPT3–GABARAPL2 binding. Next, we tested whether the association of these proteins can be enhanced with autophagy induction, and if septin-3’s levels can be manipulated with autophagy induction or inhibition, thereby confirming septin-3’s connection with autophagy.

To investigate this hypothesis, we performed experiments with human recombinant proteins, primary neuronal cell cultures and cortical slices from mice. We found that septin-3 is capable of binding LC3B, and the colocalization of these proteins can be enhanced by autophagy induction in primary neuronal cell cultures.

## Materials and methods

### Animals

C57BL/6N mice (Innovo Kft., Isaszeg, Hungary) were housed under standard laboratory conditions (24 °C, 50–60% relative humidity, 12 h light/dark cycle) with food and water ad libitum in a room maintained in a specific pathogen-free animal facility.

### Bioinformatics

We used Phyre2 server [35] for modeling the structure of septin-3. This model was subjected to MD simulations as implemented in GROMACS [36], using the AMBER-ff99SB*-ILDNP force field [37] and TIP4P parametrization [38]. The total charge of the system was neutralized, and the physiological salt concentration was set by placing Na^+^ and Cl^−^ ions. Energy minimization of starting structures was followed by sequential relaxation of constraints on protein atoms in three steps and an additional NVT step (all of 200 ps) to stabilize pressure. One microsecond trajectories of NPT simulations at 283 K and at 1 bar were recorded. Snapshots were collected at every 20 ps. For the calculation of root mean square fluctuation (RMSF) of atomic positions in the trajectory and for the conversion to B factor values, we used the GROMACS inbuilt functions for the frames in the 100–1000 ns period. Molecular graphics was performed with the UCSF Chimera package (University of California, San Francisco) [39].

### Microscale thermophoresis and ratiometric fluorescence assay

Human recombinant proteins neuronal-specific septin-3, LC3B and GABARAPL2 were purchased from Novus Biologicals (NBP2-51926, NBP1-50960 and UL-420-500, respectively). Proteins were dialyzed against phosphate-buffered saline (PBS), pH 7.4, 0.05% Tween 20, using D-tube Dialyzer Mini tubes (Merck). LC3B and GABARAPL2 were labeled using Monolith His-Tag Labeling Kit RED-tris-NTA 2nd Generation (MO-L018, NanoTemper Technologies) according to the manufacturer’s instructions. In the binding assays, septin-3 was used as a ligand in three parallel twofold dilution series, with starting concentrations 6.9 μM and 13.4 μM, in case of labeled LC3B and GABARAPL2 targets, respectively. Target concentrations were set to a constant 50 nM per dilution. Microscale thermophoresis (MST) and ratiometric measurements were carried out simultaneously on a Monolith X instrument (NanoTemper Technologies), at wavelengths 670 nm and 650 nm, with 60% (medium) MST power, within 10 min after binding assay setup. The ratiometric approach of the spectral-shift technology allows focusing the measurement on the active fraction of the target supporting higher signal-to-noise output for the demanding samples. Moreover spectral-shift measurements offer an orthogonal approach to validate for instance MST measurements if needed. Dissociation constants (*K*_D_) were defined with OriginPro (2016, OriginLab Corporation) using curve fitting on the acquired data by Hill’s equation, with Hill coefficients set to 1.

### Fluorescence polarization assay

Fluorescence polarization assay was carried out on 25 °C in PBS pH 7.4 supplemented with 0.05% Tween 20, using a Synergy HT multi-detection microplate reader (Bio-Tek). Human recombinant LC3B and GABARAPL2 (Novus Biologicals NBP1-50960 and UL-420-500, respectively) probes were labeled with Alexa Fluor 488 Microscale Protein Labeling Kit (Thermo Fisher Scientific) according to the manufacturer’s instructions. The labeled probes (LC3B and GABARAPL2) were titrated by a twofold dilution series of septin-3 ligand. The twofold dilution series of septin-3 were made with a starting concentration of 15 μM in case of SEPT3-LC3B measurements and 7 μM in case of SEPT3-GABARAPL2 measurements. The labeled probe concentrations were set to 50 nM. Direct titrations were made in three parallels. The measured polarization data was plotted against the concentration of septin-3 and fitted by OriginPro using the following equation:

$$A = A_{\min } + \left( {A_{\max } - A_{\min } } \right)\frac{{\left( {K_{{D,{\text{eq}}}} + \left[ {{\text{DL}}} \right]_{0} + \left[ {{\text{BP}}} \right]_{0} } \right) - \sqrt {\left( {K_{{D,{\text{eq}}}} + \left[ {{\text{DL}}} \right]_{0} + \left[ {{\text{BP}}} \right]_{0} } \right)^{2} - 4\left[ {{\text{DL}}} \right]_{0} \left[ {{\text{BP}}} \right]_{0} } }}{{2\left[ {{\text{BP}}} \right]_{0} }},$$

where $A_{\min }$ and $A_{\max }$ are the margins of the observed milliP scale, ${[\mathrm{DL}]}_{0}$ is the initial ligand (SEPT3) concentration and ${[\mathrm{BP}]}_{0}$ is the labeled probe concentration (constant 50 nM) and ${K}_{D,\mathrm{eq}}$ is the dissociation constant.

### Tryptophan fluorescence and quenching

Solvent accessibility of Trp residues to water was calculated using the DSSP algorithm [40], using 1.4 Å as probe radius. The accessible surface is given in Å^2^ in the output files. For the iodide ion, the GetArea server [41] was used with 2.2 Å probe radius [42]. For the evaluation, we used 51 frames of the 1 microsecond-long trajectory with 20 ns intervals. The calculated values are ratios of side-chain surface area accessible for iodide ion to the fully accessible "random coil" value per residue. Residues are considered solvent exposed if the ratio value exceeds 50% and buried if the ratio is less than 20%.

Trp fluorescence of human recombinant septin-3 (NBP2-51926, Novus Biologicals) was measured in 3 μM concentrations (in TBS, 0.05% Tween 20, pH 7.6) in a quartz micro-cell with 1.5 × 1.5 mm dimensions (105.252-QS, Hellma) using a FluoroMax spectrophotometer (SPEX Industries). Human recombinant LC3B (NBP1-50960, Novus Biologicals) was measured at 8 μM concentration (in TBS, 0.05% Tween 20, pH 7.6). Quenching of surface tryptophans was achieved by adding 200 mM NaI to the sample. Complexes were examined in a 3 μM final concentration of septin-3 and 8 μM final concentration of LC3B (in TBS, 0.05% Tween 20, pH 7.6). Emission scans were recorded in the 310–400 nm wavelength range with 0.5 nm increment using an excitation wavelength of 295 nm. 5 nm excitation and emission bandwidths were used.

Scans were analyzed with OriginPro software (2016, OriginLab Corporation), where Raman peak of buffer was subtracted from both septin-3 and LC3B measurements, and the corrected LC3B spectra were subtracted from the spectra of the complex.

### Primary neuronal cell culture (PNCC)

Pregnant mice were euthanized 18–19 days post-fertilization. Cortical brain tissues were isolated from embryonic brains in a dissecting solution (6.58 mM NaCl, 0.27 mM KCl, 9 µM Na_2_HPO_4_, 0.011 mM KH_2_PO_4_, 0.28 M HEPES, 33.3 mM d-Glucose, 43.8 mM sucrose, adapted from [43]). Minced tissues were placed in Hanks’ balanced salt solution with calcium and magnesium (HBSS + / + , 14025092; Thermo Fisher Scientific). DNase (11284932001; Sigma-Aldrich/Merck) and trypsin (15090046; Thermo Fisher Scientific) were added for 15 min at 37 °C in final concentrations 1 μg/ml and 0.035% (v/v), respectively. Neurons were triturated in a HBSS + /+ solution containing 1% (w/v) AlbuMAX I Lipid-Rich BSA (11020013; Thermo Fisher Scientific), 0.5 mg/ml trypsin inhibitor (T9003; Sigma-Aldrich/Merck), and 1 μg/ml DNase. Cell suspension was strained with 70 μm pore size strainer (734–0003; VWR) into Neurobasal media (21103049; Gibco, Thermo Fisher Scientific), containing 200 mM L-glutamine (G7513; Sigma-Aldrich/Merck), 50 × diluted B-27 supplement (17504044; Gibco, Thermo Fisher Scientific), 10 000 u/ml Penicillin–Streptomycin (15140122; Thermo Fisher Scientific) and 1 μM cytarabine (C1768; Sigma-Aldrich/Merck). Cells were plated with 1.2 × 10^5^ cells/ml (6 × 10^4^/0.5 mL) concentration onto (coverslip containing) 24-well plates, or 1.3 × 10^5^ cells/ml (4 × 10^5^/3 mL) in case of 60-mm well tissue culture plates (Greiner Bio-One). After 24 h, media was changed to cytarabine-free media. Culture media was replaced once a week for 15 days.

### PNCC treatments

Primary neurons at 15 days in vitro were treated with Autophagy Enhancer-67 (AUTEN-67; Velgene Biotechnology), bafilomycin A1 (Sigma-Aldrich/Merck; B1793), carbonyl cyanide m-chlorophenylhydrazone (CCCP, Sigma-Aldrich/Merck; C2759) dissolved in DMSO, and 3-methyladenine (3-MA, Sigma-Aldrich/Merck; L7543) dissolved in media. Dosage and time intervals were defined based on [44–47], respectively.

For AUTEN-67 treatments, medium was replaced and cells were incubated in medium containing 100 μM AUTEN-67 for 6 h. In case of the CCCP treatments, cells were incubated for 2 h in 10 μM CCCP. Control medium contained 0.001% (v/v) DMSO. For autophagy inhibition experiments, cells were treated simultaneously with 100 μM AUTEN-67, with either 100 nM bafilomycin A1 or 10 nM 3-MA, for 6 h.

For all treatment conditions, three independently prepared cultures were used (as biological triplicates).

### PNCC immunolabeling, light microscopy, and colocalization analysis

For colocalization analysis, two AUTEN-67 treated, two CCCP treated and four corresponding control wells were examined (as technical duplicates) from each culture (Fig. 5A). After treatments, cells were fixed with 4% paraformaldehyde for 5 min, then washed with PBS and stored in PBS, 0.02% Na-azide until use. For antigen retrieval, coverslips were soaked in ice-cold acetone for 10 min at − 20 °C and then washed with PBS. After blocking with 3% BSA in PBS for 1 h, plates were incubated with primary antibody solution O/N at 4 °C in 1% BSA, 0.06% Triton-X 100 in PBS. Diluted primary antibodies were as follows: rabbit anti-LC3B, 1:200 (2775, Cell Signaling Technology); mouse anti-SEPT3, 1:50 (sc-74431, Santa Cruz) and goat anti-PINK1, 1:200 (STJ71249, St. John’s Laboratory). Secondary antibody staining was done at room temperature with Cy3-conjugated anti-rabbit, 1:200 (Jackson ImmunoResearch; 711–165-152); Alexa Fluor 488-conjugated anti-goat, 1:200 (Jackson ImmunoResearch; 705–545-147) and Alexa Fluor 647 conjugated anti-mouse, 1:200 (Jackson ImmunoResearch; 715–605-151) antibodies in PBS for 1 h. We also implemented nuclear staining with DAPI, 0.1 μg/mL in PBS (D9542, Sigma-Aldrich/Merck) for 10 min.

Zeiss LSM 880 (Carl Zeiss) confocal microscope was used for image capturing with 63 × magnification (using a Plan-Apochromat 1.4NA DIC M27 objective, Carl Zeiss). Abundant A488 (septin-3) signal containing 101.4 × 101.4 μm areas were sampled for colocalization analysis. Three images were captured for each coverslip, resulting in 18 images per condition. To analyze the images, Fiji/ImageJ [48] and its plugins were used. For detection of local maxima (peaks) and segmentation of the signals (spots), we used 3D ImageJ suite [49] plugins 3D Maxima finder and 3D spot segmentation, respectively, enabling accurate and automated image processing. We used JACoP [50] ImageJ plugin for detecting colocalization based on the distance between the spots’ centroids.

One-way ANOVA was calculated to prove the nonsignificant difference between three cultures in a group (AUTEN-67 treated; AUTEN-67 treatment control; CCCP treated; CCCP treatment control). Kolmogorov–Smirnov test was used to determine the normality of the data within a group. The significance of the differences between groups was calculated with independent, two-tailed Student’s *t*-test.

To ensure that the observed colocalizations are not accidental, we simulated the colocalization with randomly placing the same centroids on the images using MATLAB (MathWorks, Inc. USA).

### Western blot

Protein lysates were prepared per culture from AUTEN-67 treated, AUTEN-67 + bafilomycin A1 treated, AUTEN-67 + 3-MA treated and control cells, grown in 60 mm cell culture plates. 100 μL thiourea/urea lysis buffer (20 mM Tris, 7 M urea, 2 M thiourea, 5 mM Mg(Ac)_2_, 4% CHAPS, pH 8.5) supplemented with 100 × diluted protease inhibitor cocktail (P8340, Sigma-Aldrich/Merck) was added to each well. Cells were scraped off onto solution with a plastic cell scraper, and, using sonication, were further homogenized. Fifteen μg protein per sample was resolved on a 20% polyacrylamide gel, and then transferred to an Amersham Hybond LFP PVDF membrane (GE Healthcare). We used the following primary and secondary antibodies and dilutions for immunostaining: mouse anti-SEPTIN3 monoclonal IgG_2b_ 1:500 (sc-74431, Santa Cruz Biotechnology); rabbit anti-β-actin monoclonal IgG, 1:50 000 (AC026, ABclonal); rabbit anti-LC3B polyclonal IgG, 1:500 (L7543, Sigma-Aldrich); rabbit anti-P62/SQSTM1 polyclonal IgG, 1:500 (P0067, Sigma-Aldrich); Alexa Fluor 488-conjugated donkey anti-rabbit, 1:800 (Jackson ImmunoResearch; 711-545-152); Alexa Fluor 647-conjugated donkey anti-mouse, 1:800 (Jackson ImmunoResearch; 715-605-151). Membranes were scanned with a TyphoonTRIO + scanner (GE Healthcare), densitometric analysis was performed using Fiji/ImageJ [48]. Signal intensities were normalized to the highest observed signal, and final values were normalized to that of β-actin. For statistical comparison, Student’s independent two-tailed *t*-test was used.

### Postembedding immunocytochemistry and electron microscopy

Acute slices with 1 mm thickness were cut from mouse cortices, and treated with 100 μM AUTEN-67 dissolved in carbogenated (95% O_2_, 5% CO_2_) artificial cerebrospinal fluid (aCSF: 125 mM NaCl, 2.5 mM KCl, 2.0 mM MgCl_2_, 1.125 mM NaH_2_PO_4_, 25 mM NaHCO_3_, 25 mM glucose, 1 mM CaCl_2_, pH 7.4) for 6 h. Control slices were kept alive in carbogenated aCSF only. Slices were fixed with 3.2% formaldehyde, 1% glutaraldehyde and 3 mM CaCl_2_ in 0.1 M sodium-cacodylate buffer pH 7.4 for 2 days. Samples were cut to smaller 1 × 1 mm blocks for further processing.

Quenching of free aldehyde was carried out by incubating the sample in 50 mM ammonium-chloride and 50 mM glycine in 0.1 M phosphate buffer (PB). The samples were post-fixed with 1% uranyl-acetate in 0.05 M maleate buffer. Dehydration was achieved using increasing ethanol concentrations by decreasing temperatures (25% for 10 min at 4 °C, 50% for 10 min at 4 °C, 70% for 10 min at − 20 °C, 96% for 20 min at − 20 °C and absolute for 60 min at − 20 °C).

Samples were soaked in LR White without benzoyl peroxide (BPO) for 24 h, and then with BPO for 48 h in UV radiation at 4 °C. From the polymerized block, ultrathin sections were cut and placed on formvar/carbon-supported nickel grids [51].

Immunolabeling was carried out on droplets placed on parafilm-wrapped plates in a humidified chamber. Grids were pre-treated at room temperature as follows: 5% H_2_O_2_ (in ddH_2_O) for 1 min; 0.1% NaBH_4_ in Tris-buffered saline (TBS) pH 7.6 for 10 min; 50 mM glycine in TBS for 30 min. Blocking was carried out using 10% fetal calf serum (FCS) in TBS for 30 min.

Primary antibodies mouse anti-SEPT3 polyclonal IgG, 1:20 (MABN1530, Merck-Millipore) and rabbit anti-LC3B polyclonal IgG, 1:20 (L7543,) were used in 5% FCS in TBS with overnight incubation at 4 °C. Ten nm colloidal gold-conjugated polyclonal goat anti-mouse secondary IgG, 1:50 (G7652, Sigma-Aldrich) and 6 nm colloidal gold-conjugated polyclonal anti-rabbit secondary IgG, 1:50 (711–195-152, Jackson ImmunoResearch) were used in 2% FCS in TBS for 90 min at room temperature. After washing, specimens were post-fixed with 1% glutaraldehyde in TBS for 10 min.

Five AUTEN-67 treated and 5 control grids were also immunolabeled for septin-3 for quantification, where Kolmogorov–Smirnov test of normality and Mann–Whitney *U* test were used.

Electron microscopy images were taken with a JEM-1011 transmission electron microscope (JEOL, USA).

## Results

### Atg8 interacting motifs (AIMs) of septin-3

**Fig. 1:**
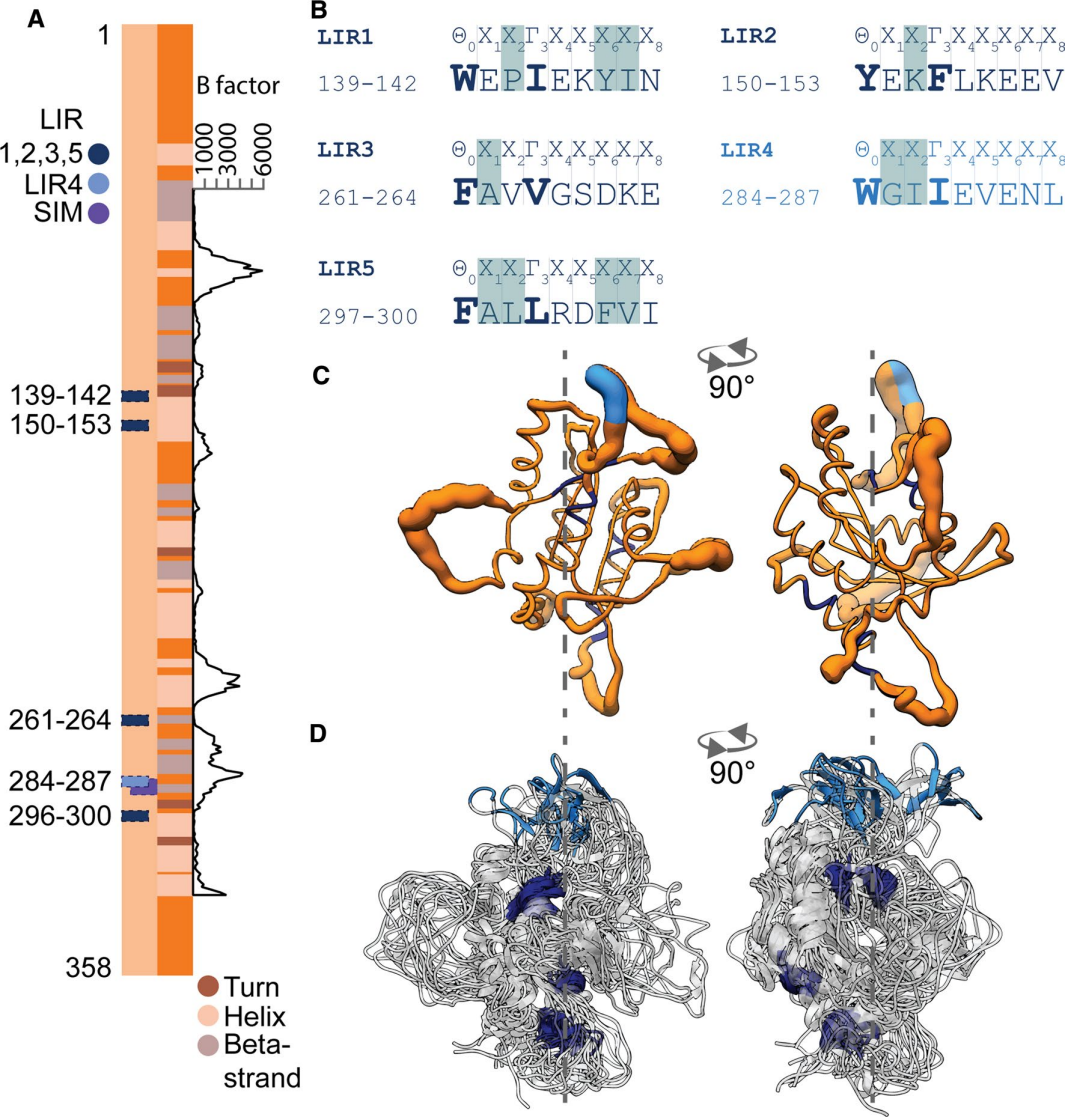
Sequential and structural location of LIR motifs in septin-3. **A** Positions of five ELM predicted LIR motifs on the canonical human SEPT3 A chain, with secondary structure display. The most likely functional LIR4 is highlighted with light blue, while LIR1, 2, 3, and 5 are shown in dark blue. LIR4-overlapping SUMO interacting motif (SIM) is marked with purple. B factor analysis scale of residues 63–329 reveals the highly mobile loop regions of the molecule. **B** ELM Predicted LIR motif sequences of SEPT3 by the canonical LIR motif *θ*_0_-*X*_1_-*X*_2_-Γ_3_, where θ marks an aromatic, Γ an aliphatic, and X an arbitrary residue. Θ and Γ residues are shown in bold, other key residues determining interactions or functionality (described in the text) are shown with grey background. **C**, **D** Molecular dynamics (MD) simulations of septin-3 structure. **C** Septin-3 structural mobility is visualized by B-factor representation. LIR4 motif is located at a highly flexible, exposed region of the molecule (light blue). **D** Superposition of representative structure ensemble from MD trajectory. LIR motifs are colored as in (**A**)

Mapping septin-3’s functional motifs seemed a promising start to explore its interactions and to shed light on its probable functions in the synapse. This way, using the ELM database [52], AIMs were predicted in septin-3’s sequence, regardless of its cellular localization. AIMs can be LC3-interacting regions (LIRs) or GABARAP-interacting motifs (GIMs), but in the literature, LIR is commonly used as a synonym for AIM and the binding specificity (either LC3 or GABARAP) is described later. The canonical human septin-3 owns five potential LIR motifs in its primary structure. Predicted motifs can be found in septin-3 isoforms A and B but not C. Predicted core LIR motifs can be found at positions 139–142, 150–153, 261–264, 284–287, and 297–300, denoted here as LIR1-5, respectively (Fig. 1A, B). For these motifs, the conservation scores are 0.95, 1.00, 0.06, 1.00, and 1.00, respectively, which means, except for LIR3, they could have conserved functions [53]. Based on extensive research on other LIR (AIM) motif-containing Atg8 binding proteins and binding specificities [54, 55], the following predictions can be made for septin-3. Hydrophobic residues (tyrosine, isoleucine) in position X6 and X7 of LIR1 could assist GABARAP binding instead of LC3; however, proline in X2 is highly disruptive to both Atg8 homologs’ binding, therefore, this LIR is hardly functional. Lysine in X2 position of LIR2 would probably inhibit LC3 but tolerates GABARAP binding. LIR3 has a very low conservational score (0.06). Alanine in position X1 would weaken the interaction with Atg8. LIR5 also lines up an alanine in position X1, but has an aliphatic leucine in X2, that affirms LC3 binding. Phenylalanine and valine in positions X6 and X7 favor GABARAP binding. LIR4 could highly favor LC3 binding, containing aliphatic leucine in both X2 and Γ3 positions (Fig. 1B).

Results of the B-factor analysis and its structure ensemble from MD simulations (Fig. 1C, D) show that flexibility of LIR4 is conspicuous, while the other motifs are located in rigid and mostly buried regions. These results further strengthen the possibility of LIR4’s functionality, as it has properties suitable for accessing the binding site at Atg8.

Another striking prediction of ELM is a SUMO-binding motif overlapping with LIR4 (Fig. 1A). This raises the possibility of LIR4 modulation by noncovalent SUMO binding, since septin-3 associates with every protein of the SUMO-ligation complex in the brain, namely: SUMO ligase PIAS3, SUMO conjugating enzyme UBE2I and SUMO1 [34].

### Septin-3 binds to LC3B in vitro with a sub-micromolar *K*_D_

**Fig. 2:**
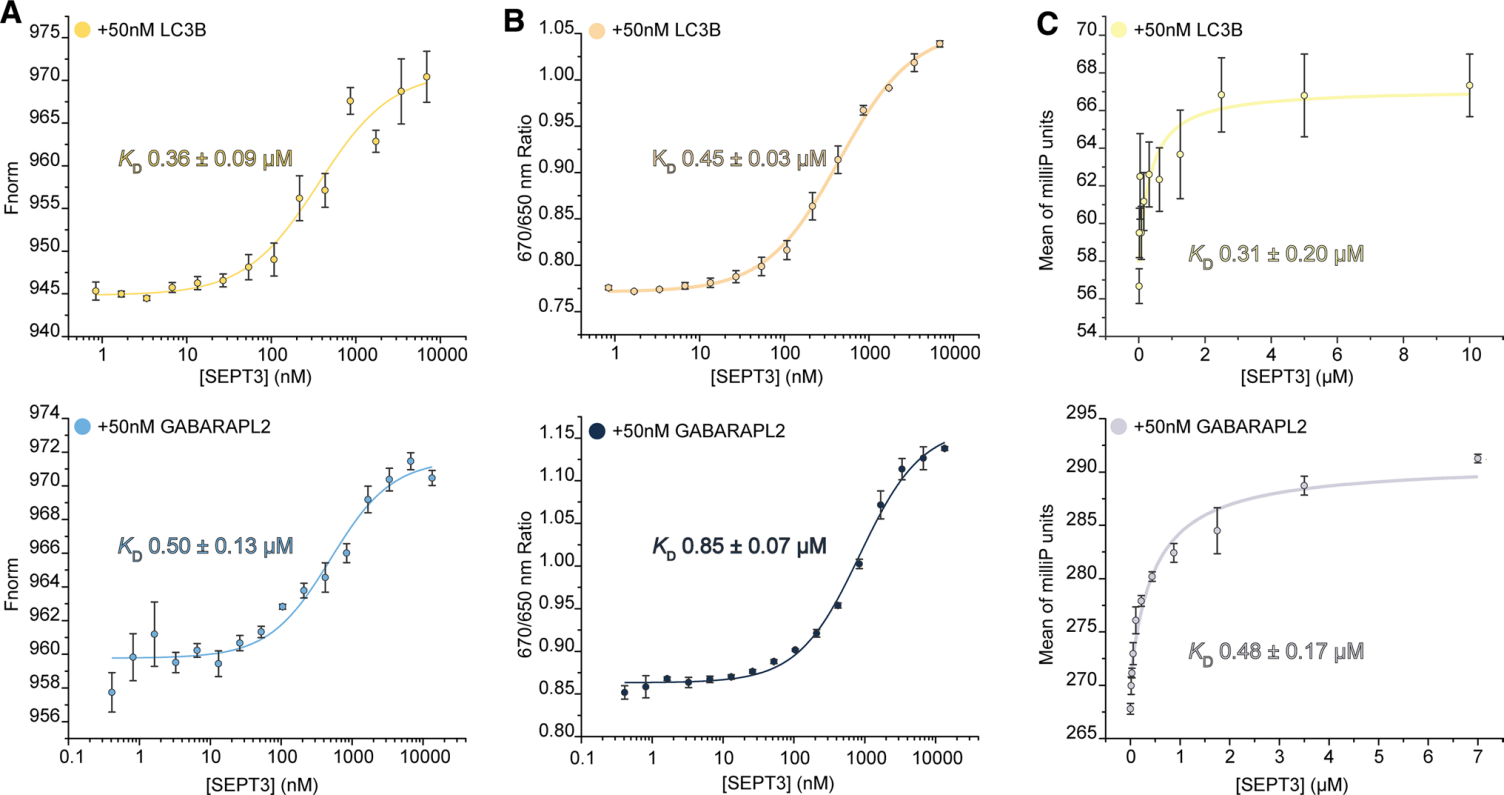
Protein–protein interaction measurements of septin-3 (SEPT3) and Atg8 homologs. **A** Microscale thermophoresis dose–response curves of LC3B-SEPT3 (yellow) and GABARAPL2-SEPT3 interactions (blue). *X* axis represents SEPT3 concentrations on a log scale, while *Y* axis reflects MST dose–response, normalized to baseline (Fnorm). Corresponding *K*_D_ values and *K*_D_ confidence are displayed. **B** Spectral shift fluorescence response of LC3B-SEPT3 (yellow) and GABARAPL2-SEPT3 (blue). **C** Fluorescence polarization direct titration curves of LC3B-SEPT3 (marked yellow) and GABARAPL2-SEPT3 (marked blue) interactions. *X* axis represents SEPT3 concentrations on a linear scale, while *Y* axis reflects polarization

To test the hypothetical binding of LC3B, and compare that to the already reported GABARAPL2 binding by septin-3 [34], commercially available recombinant human septin-3 and Atg8 homologs LC3B and GABARAPL2 were used in microscale thermophoresis and fluorescence polarization experiments. MST measurements carried out with septin-3 and LC3B confirmed the interaction with a dissociation constant 362.3 ± 96.5 nM (*K*_D_ ± SEM), by curve fitting on the means of three parallel measurements. MST of septin-3 with GABARAPL2 resulted a similar *K*_D_ of 496.7 ± 127.6 nM (*K*_D_ ± SEM) (Fig. 2A).

These interactions were further verified by the spectral shift technology of the Monolith X instrument. Interaction of the molecules leads to the change of the chemical environment around the fluorescently labelled target. This change in the fluorescently labeled target environment results in a red-blue shift, or broadening of the fluorescence emission peak. Fluorescence is recorded and a 670 nm/650 nm ratio is calculated and plotted against the ligand concentration.

Ratiometric analysis resulted in *K*_D_ values 445.1 ± 28.5 nM and 847.5 ± 74.4 nM for septin-3–LC3B and septin-3–GABARAPL2, respectively (Fig. 2B).

To further validate these results, an additional fluorescence polarization assay experiment was carried out and resulted in a very similar range of dissociation constants as it was observed in MST measurements. For septin-3–LC3B a binding constant of 312.9 ± 199.8 nM (*K*_D_ ± SEM), while for septin-3–GABARAPL2, 478.5 ± 169.6 nM (*K*_D_ ± SEM) were calculated (Fig. 2C).

In conclusion, septin-3 binds both Atg8 homologs with a sub-micromolar *K*_D_, which imply moderate affinity of septin-3 for LC3B and GABARAPL2.

### Binding of LC3B partly saves septin-3’s surface Trp fluorescence from quenching

**Fig. 3:**
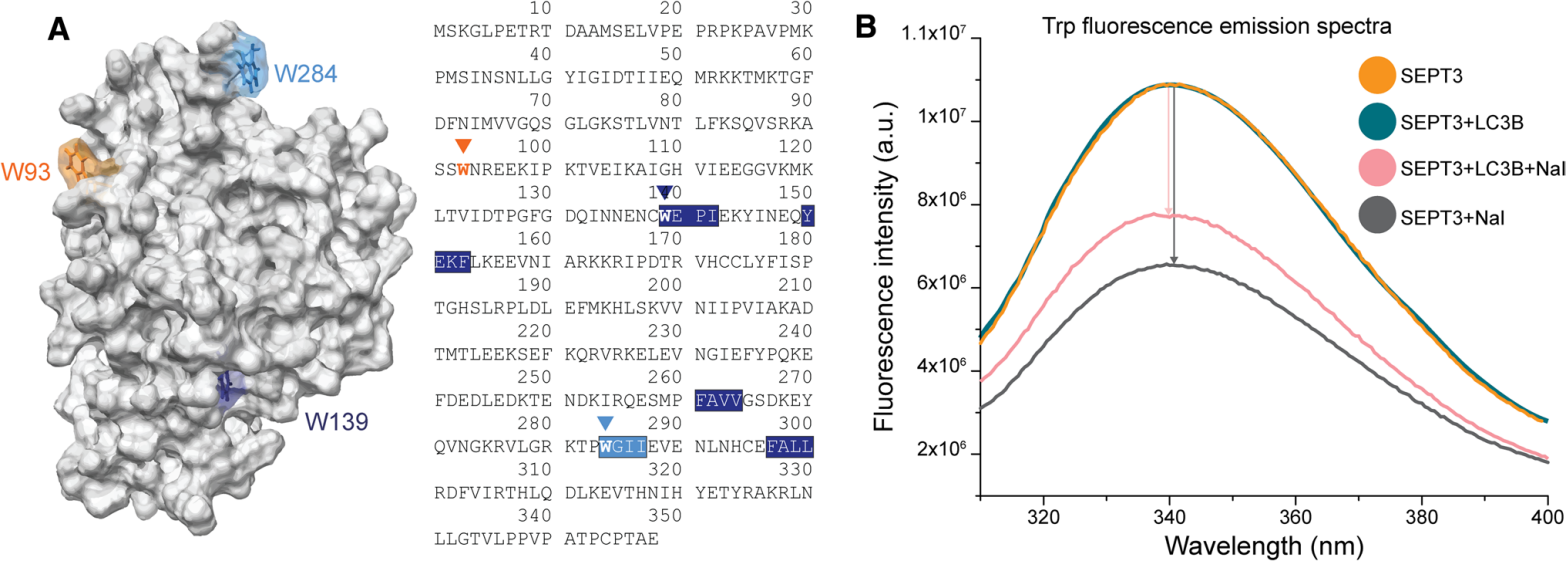
Trp fluorescence of septin-3. **A** Structure and sequence of septin-3, marking positions of tryptophans. Core LIR4 and corresponding W284 is marked light-blue, while LIR1 W139 is marked dark-blue. Non-LIR W93 is marked orange. **B** Trp fluorescence spectra of septin-3 (orange), septin-3-LC3B complex (turquoise), septin-3 after adding 200 nM NaI (grey), septin-3 in complex with LC3B after adding 200 nM NaI (pink)

We wanted to observe the effect of LC3B binding on the quenchability of septin-3 surface tryptophans. LC3B does not contain Trp, while septin-3 has three tryptophans in its sequence, W93, W193 and W284. Two of them, W139 and W284, are located in predicted LIR motifs, LIR1 and LIR4, respectively. Large anions, such as iodide, can be used to selectively quench fluorescence of surface Trps, as they cannot penetrate into hydrophobic protein structures [56]. Based on molecule models and MD simulation results, W139 of LIR1 is buried, while W93 and W284 of LIR4 are on the surface and are highly mobile (Figs. 3A, S1A). Solvent accessibility of Trp residues were calculated on MD models using DSSP [40] for water and GETAREA [41] for iodide. Results for water are 162.24 ± 58.42, 48.86 ± 18.82 and 203.00 ± 25.70 Å^2^ (mean ± SD) for W93, W139 and W284, respectively. Probe radius for water was 1.4 Å, while for iodide it was 2.2 Å [42]. Percentage accessibilities for iodide, compared to a fully exposed “random coil” structure are 70.7 ± 29.3%, 14.1 ± 8.4%, 82.8 ± 17.6% for W93, W139 and W284, respectively (Fig. S1C). The residue is considered buried under 20%, and exposed over 50%. This means W139 is indeed inaccessible for iodide, compared to W93 and W284 (*p* < 1 × 10^–7^) (Fig. S1B). We measured the intrinsic Trp fluorescence spectra of septin-3 and repeated it after selective quenching with NaI. When LC3B was added to septin-3 solution before adding iodide, the drop in Trp fluorescence was reduced, indicating that LC3B protected Trp from iodide quenching (Fig. 3B). Without the quencher, adding LC3B did not change significantly the fluorescent spectra of septin-3, supporting that the different drops in intensity are caused by the different accessibility of the quencher. Therefore, a Trp is involved in septin-3’s LC3B binding. Because, to our knowledge, LC3B binding is realized through LIR-Atg8 interaction, and, in case of LIR independent binding, through ubiquitin interacting motifs (UIMs) [57] (which cannot be found in septin-3’s sequence based on PROSITE scan [58]), LIR4 W284 is consequently engaged in LC3B binding.

### Colocalization of septin-3 and LC3B increased due to autophagy induction in primary neuronal cultures

**Fig. 4:**
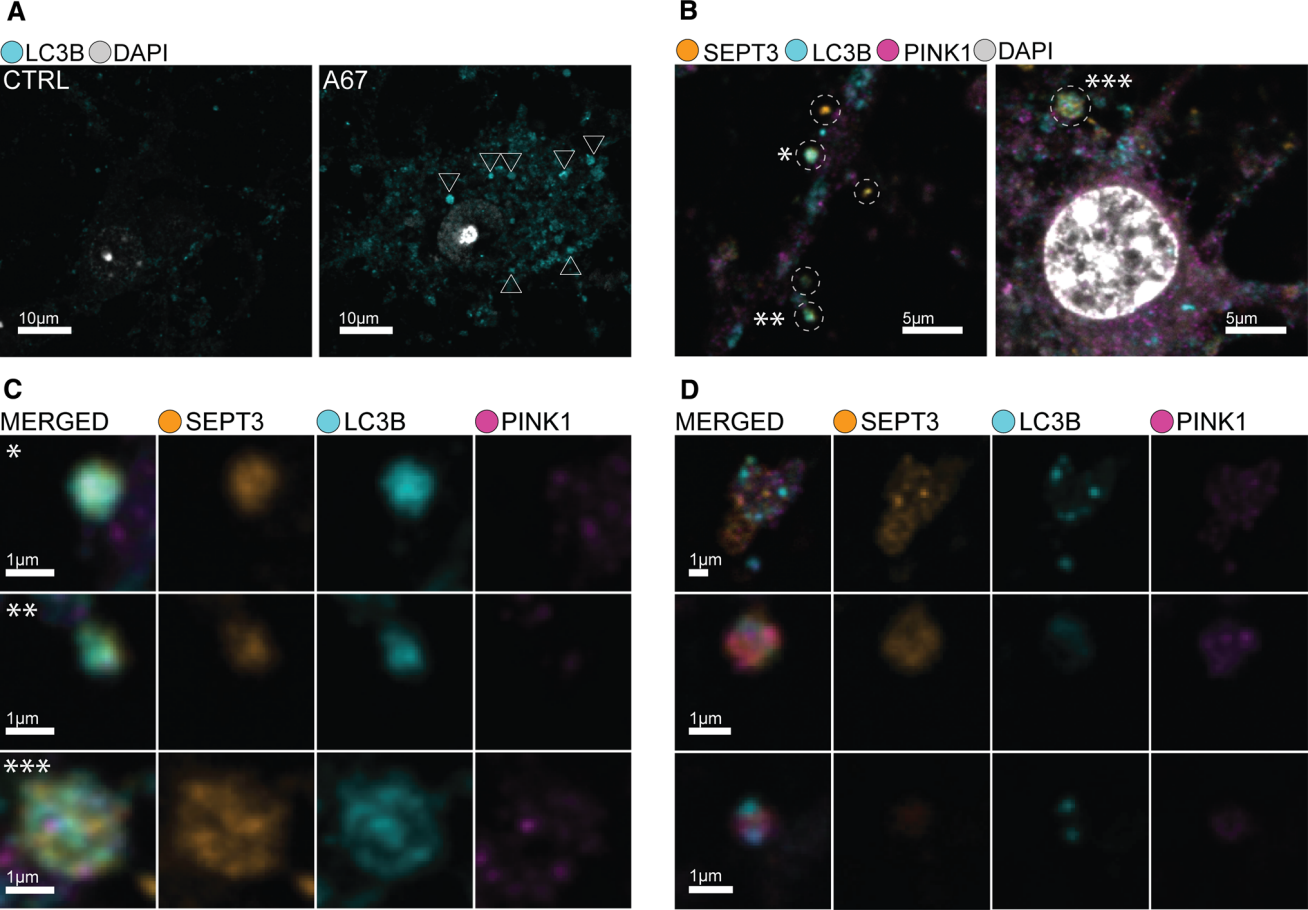
Confocal microscopy images of primary neuronal cells. **A** Representative image of enriched LC3B positive particles in the cell body of an AUTEN-67 treated primary neuron indicate enhanced autophagy compared to control. **B** Overlapping spots of fluorescent signals along the axons (left) and in the cell body (right). **C** Enlarged and resolved images of overlapping signals from B, with observable LC3B and septin-3 (SEPT3) colocalization, where SEPT3’s colocalization with PINK1 is minimal. However, more complex colocalization patterns of LC3B–PINK1–SEPT3 can be observed from other parts of the sample (**D**)

After proving the feasibility of the interaction of septin-3 and LC3B in vitro, we investigated the two proteins’ colocalization in neurons. Primary neuronal cells were cultured for 15 days to allow for sufficient synapse formation [59]. We used immunostaining for the autophagosome marker LC3B to investigate its colocalization with septin-3 after chemically-induced autophagy. For this, we used the drug AUTEN-67, which has already been proven to induce autophagy in primary neuronal cells [44]. The administration of AUTEN-67 induced visible accumulation of LC3B positive particles in the cell bodies of neurons compared to untreated ones (Fig. 4A). Immunocytochemistry for septin-3 and LC3B resulted in observable colocalization of the two proteins in the treated samples along the axons and in the cell bodies (Fig. 4B, D).

**Fig. 5:**
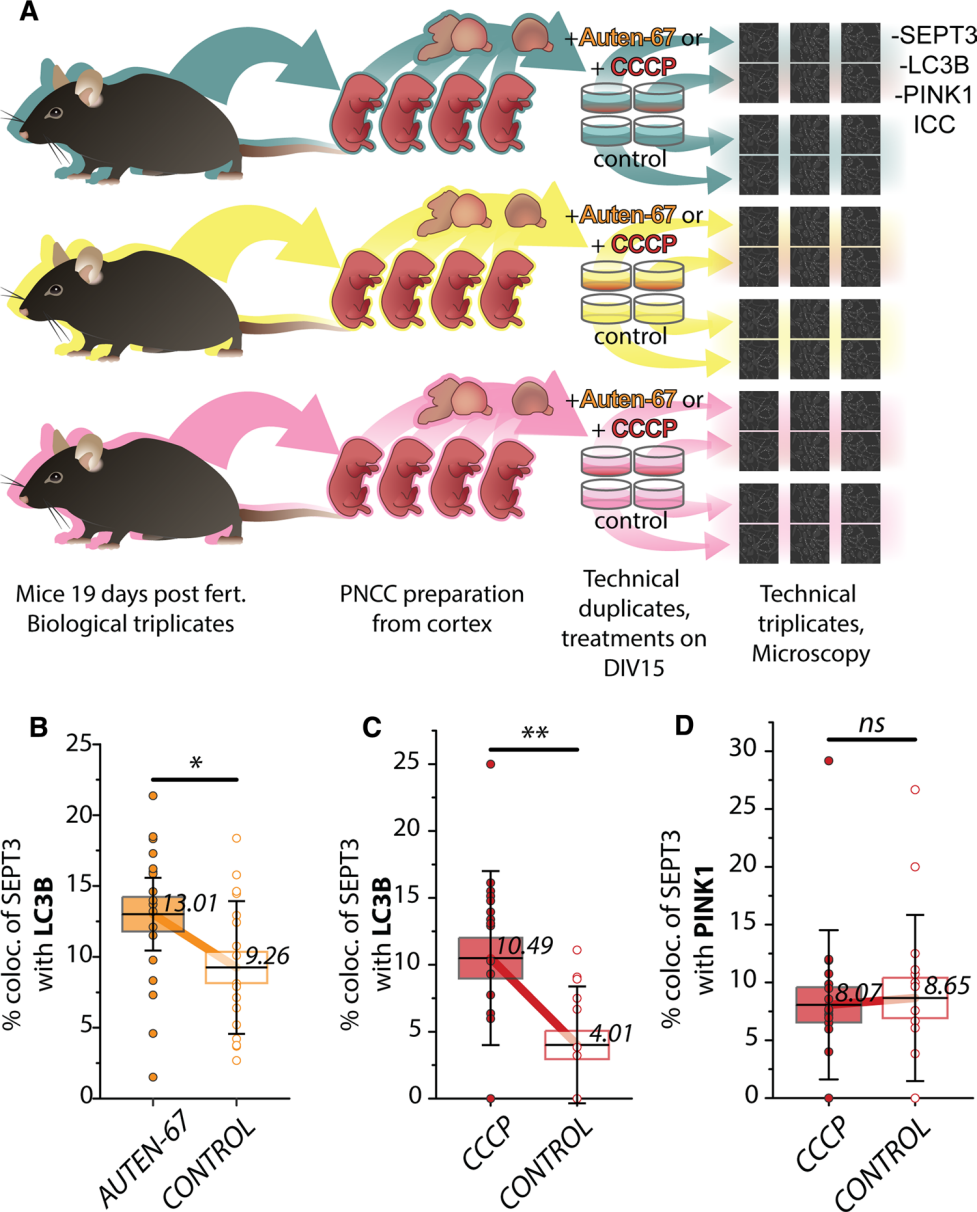
Experimental setup and results of colocalization analyses of septin-3 (SEPT3) due treatments. **A** Embryos were excised from three different dams to create biological triplicates of cultures. Embryonic cortices were isolated and homogenized to prepare cortical primary neuronal cell cultures. Two wells per culture were exposed to treatments (either AUTEN-67 or CCCP), two wells per culture were used as control. Cells were immunostained against SEPT3, LC3B and PINK1. Three images were captured and analyzed per well for every treatment/control condition. **B**–**D** Percentage comparison of colocalizing signals between treated and control cells. Means are marked with horizontal lines with values; boxes represent standard error of the mean (SEM). Flags indicate standard deviation (SD). In the figure legend, results are presented as mean ± SEM; SD. The *p*-values are of independent two-tailed Student’s *t*-test. **B** LC3B colocalizing SEPT3 in AUTEN-67 treatments, 13.01% ± 1.21%; 5.14%. Controls: 9.26% ± 1.1%; 4.68%. Significance: *p* = 0.0283. **C** LC3B colocalizing SEPT3, CCCP treatments: 10.49% ± 1.53%; 6.50%. Controls: 4.01% ± 1.06%; 4.38%. Significance: *p* = 0.0016. **D** PINK1 colocalizing SEPT3, CCCP treatment: 8.07% ± 1.52%; 6.46%. Controls: 8.65% ± 1.74%; 7.19%. Significance: *p* = 0.80

Two wells were treated with AUTEN-67 and two were used as controls in each of the three cultures. Three images were captured per well, resulting in 18–18 confocal images of the AUTEN-67 treated and control cells. Quantification of the colocalization confirmed that septin-3’s joint appearance with LC3B is considerably higher in autophagy-enhanced cells (13.01% ± 1.21% (mean ± SEM) of the total septin-3 signal), compared to the untreated ones (9.26% ± 1.1% (mean ± SEM)) (*p* = 0.03) (Fig. 5B). As a reference, we simulated the colocalization by randomly placing the objects on the images resulting in 0.26% and 0.24% colocalization for AUTEN-67 treated and control samples, respectively, excluding that our result is just a coincidence.

### PINK1 and septin-3 colocalization was unaltered, while septin-3–LC3B colocalization increased due to CCCP treatment

C1q-tagged synapses show signs of mitochondrial oxidative stress [31]. Also, flawed mitophagy, mitochondrial fission, and autophagosome accumulation are well-known phenomena in AD pathogenesis, whereas, by stimulating mitophagy, memory impairment can be reversed [60–62]. Mammalian septins in fact play role in mitochondrial fission and crosslink mitochondria and the pathogen *Shigella* to help the pathogen’s clearance through mitophagy [63]. Because of the aforementioned correlations, we proposed that septin-3 may play role in mitophagy by binding to mitochondria in dysfunctional synapses. PTEN-induced putative kinase 1 (PINK1) is imported to the mitochondrial inner membrane continuously, where it gets cleaved and then degraded in the cytosol. Faulty import or inner membrane depolarization causes PINK1 accumulation on the outer membrane of mitochondria [64], therefore, PINK1 can be used as an early mitophagy marker. CCCP, a weak acid acts as an uncoupling agent, causes membrane depolarization, and induces mitophagy [65, 66]. We used CCCP treatment on primary neuronal cells to induce mitophagy, and performed immunocytochemistry against septin-3, PINK1 and LC3B. Based on our imaging experiments, CCCP treatment did not result in an enhanced co-association of SEPT3 to PINK1: 8.07% ± 1.52% (mean ± SEM) of the total SEPT3 signal colocalized with PINK1 in treated cells and 8.65% ± 1.74% (mean ± SEM) in the untreated controls (*p* = 0.80) (Fig. 5D). On the other hand, septin-3 to LC3B colocalization increased significantly (*p* = 0.002) in CCCP treated cells, compared to untreated ones (10.49% ± 1.53% (mean ± SEM) vs. 4.01% ± 1.06% (mean ± SEM)) (Fig. 5C). Simulation of placing the objects randomly on the images resulted in 0.3% and 0.2% colocalization suggesting that our results are valid and not a coincidence.

Due to the result that colocalization of septin-3 to PINK1 remained unchanged (Fig. 5D), and since PINK1 accumulates early on the dysfunctional mitochondria, we conclude that septin-3 may not be involved in the mitochondria elimination of the synapse as a mitophagy receptor in the PINK1-Parkin pathway. However, like in the case of AUTEN-67 treatment, the localization of septin-3 to LC3B increased (Fig. 5C). This presumes that the formed, probably mitochondria-containing autophagosomes are tagged with septin-3. It worths mentioning, that septin-3, being a presynaptic protein, is underrepresented in fluorescent spots compared to abundant PINK1 and LC3B, with an order of magnitude. Therefore examining the changes in PINK1 and LC3B colocalized septin-3 signal is of higher priority. However, ~ 1 percent significant change can be detected in PINK1 and LC3B signals that colocalize with septin-3 in CCCP treatment, which means an average of ten times more autophagic structures on the images compared to controls (Fig. S2).

### Septin-3 protein levels change accordingly to LC3B-II and P62 in autophagy enhancement and inhibition

Bafilomycin A1 antibiotic treatment blocks the late phase of autophagy, as it induces the accumulation of undigested autolysosomes by blocking V-ATPases and preventing lysosomal acidification. The drug also contributes to diminishing the autophagosome-lysosome fusion by disrupting Ca^2+^ gradients through Ca^2+^ ATPase SERCA inhibition [67]. Therefore, an elevation can be detected in protein levels of the autophagic machinery, especially in the truncated, membrane associated form of LC3B (LC3B-II), due to treatment. On the other hand, 3-methyladenine (3-MA), a phosphatidylinositol 3-kinase (PI(3)K) inhibitor blocks the formation of the phagophore, by inhibiting class III PI(3)K Vps34/PIK3C3 [68–70]. If autophagy is blocked in this early phase, no accumulation of the membrane-bound LC3B-II is expected.

Protein levels/expression of septin-3, LC3B-II and a known selective autophagy receptor, p62/SQSTM1 [71], was observed with western blot at molecular weights of ~ 40, 16 and ~ 62 kDa, respectively. Protein levels in AUTEN-67 treated primary neurons, without inhibitors, and in the presence of bafilomycin A1 or 3-MA, were compared to untreated controls. Treatments were carried out on three cultures, originated from three animals.

**Fig. 6:**
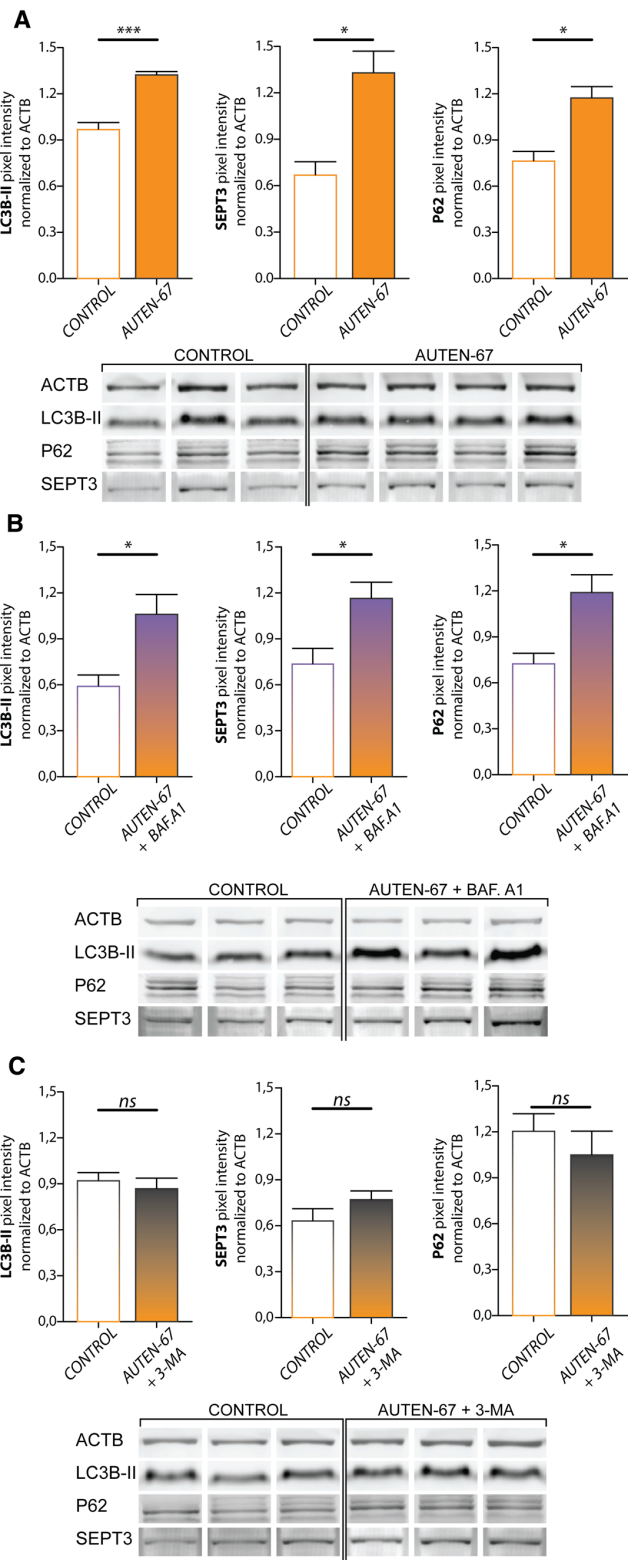
Western blot analysis of LC3B, septin-3 and p62 protein level changes in primary neurons presented as mean ± SEM due to treatments, with corresponding bands. **A** Effect of 100 μM AUTEN-67 treatment compared to untreated controls: significant changes in levels of LC3B-II (*p* = 0.0006), septin-3 (*p* = 0.014) and p62 (*p* = 0.010) can be observed. **B** Effect of 100 μM AUTEN-67, 100 nM Bafilomycin A1 treatment compared to untreated controls: significant changes in levels of LC3B-II (*p* = 0.034), septin-3 (*p* = 0.043) and p62 (*p* = 0.026) can be observed. **C** Effect of 100 μM AUTEN-67, 10 nM 3-MA treatment compared to controls: no significant change in protein levels can be observed (*p* = 0.586 for LC3B-II, *p* = 0.231 for septin-3 and p = 0.0468 for p62)

AUTEN-67 treatment alone induced a significant increase in levels of LC3B-II (*p* = 0.0006), as well as of septin-3 (*p* = 0.014) and p62 (*p* = 0.010) (Fig. 6A). Emerging levels of septin-3 due to autophagy enhancement excludes that septin-3 is an autophagy cargo prone to be degraded. Bafilomycin A1 treatment alongside with AUTEN-67 also led to a significant increase in levels of LC3B (p = 0.034), septin-3 (*p* = 0.034) and p62 (*p* = 0.026) (Fig. 6B). 3-MA with AUTEN-67 treatment did not induce significant changes in levels of LC3B (*p* = 0.586), septin-3 (*p* = 0.231) or p62 (*p* = 0.468) (Fig. 6C).

From this result we conclude, that enhanced autophagy induces elevation in septin-3 levels, and autolysosome inhibition causes septin-3 accumulation. Septin-3 levels change similarly to selective autophagy marker p62’s levels in treatments.

### Septin-3 is present on mitochondria and autophagic, LC3B positive structures on electron micrographs

**Fig. 7:**
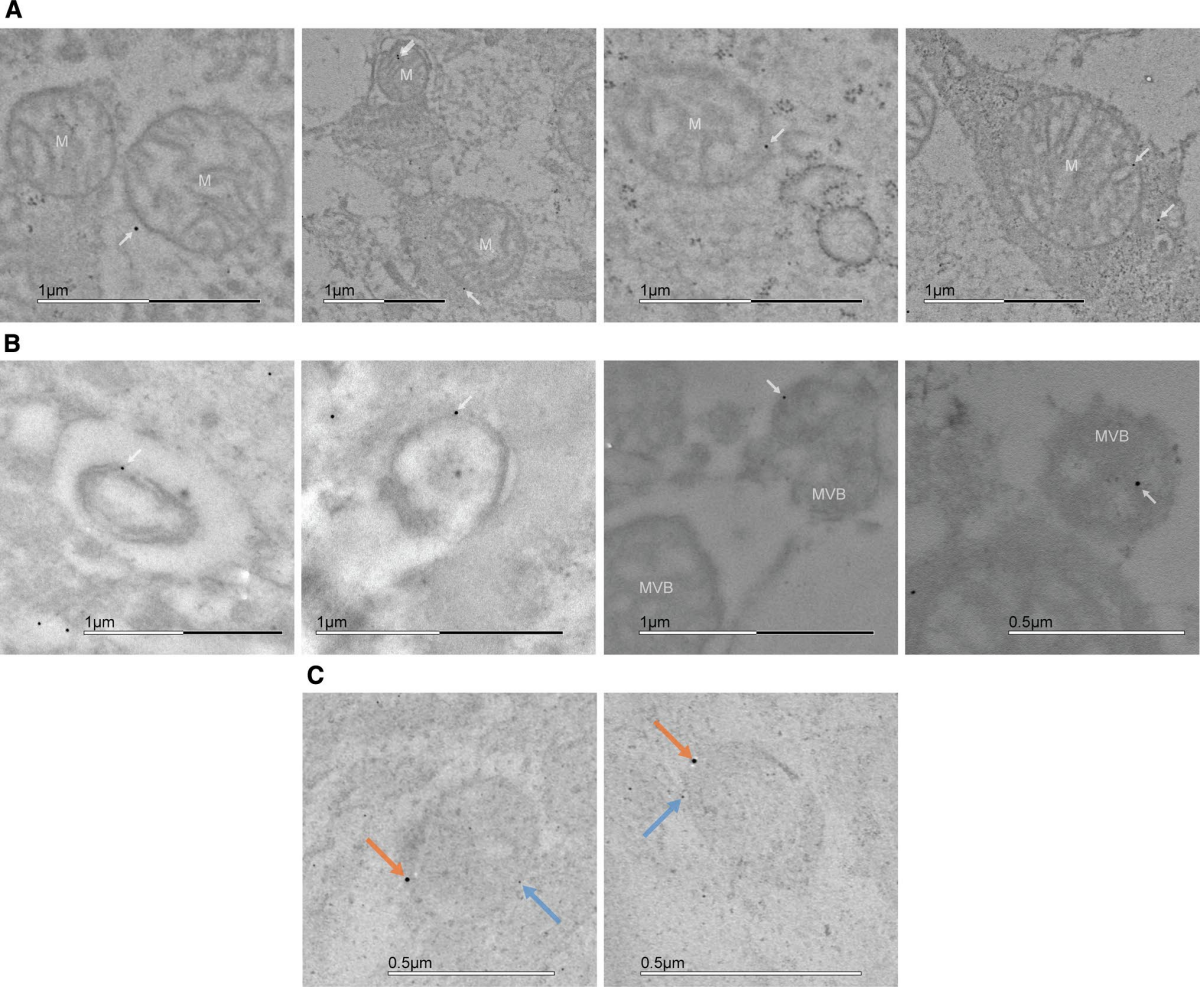
TEM images of immunostained cortical samples. **A** 10 nm colloidal gold stained septin-3 localizing to mitochondria (M). **B** Septin-3 located at double-membrane and multivesicular body-like (MVB) structures. **C** Septin-3 located on LC3B positive autophagic vesicles with double staining of septin-3 (orange arrows) and LC3B (blue arrows), using 10 nm and 6 nm colloidal gold, respectively

To examine if SEPT3–LC3B interaction is localized to autophagic vesicle membranes (instead of, e.g., cytoplasmic LC3B aggregates), we embedded AUTEN-67 treated and untreated cortical brain slices for transmission electron microscopy (TEM). On average, significantly more colloidal gold can be observed in AUTEN-67 treated samples, compared to controls (Fig. S4A), that correlates with the observed elevation in septin-3 levels in AUTEN-67 treated primary neurons (Fig. 6A). In addition to the presynaptic site (Fig. S3A), septin-3-representing gold particles are mainly found on membranes and dense cellular structures, but are present on microtubules also (Fig. S3B). Despite the result that septin-3’s localization to the early mitophagy marker PINK1 unchanged during mitophagy, septin-3 was found on mitochondria, however, the number of septin-3 positive mitochondria per micrograph remained unchanged when compared autophagy enhanced and control samples (Fig. S3B). Nanogold particles can be detected on autophagosome-, autolysosome-, and multivesicular-body-like double-membraned structures, also (Fig. 7B). To ensure that these structures were autophagy related, septin-3 and LC3B double-labeled grids were examined. Both septin-3 and the autophagosome marker can be observed on organelles (Fig. 7C).

**Fig. 8:**
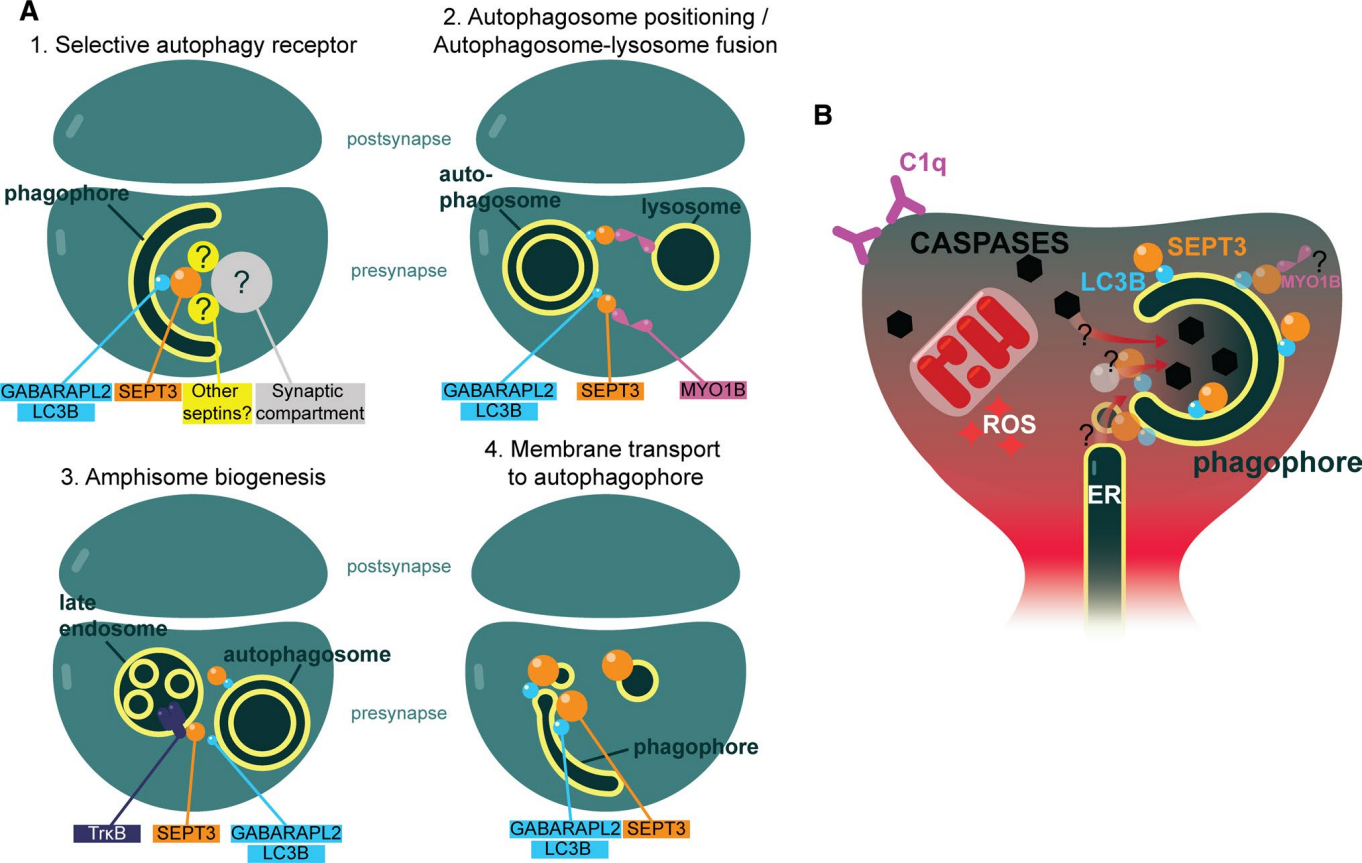
Theories of septin-3 function. **A** Hypothetical roles of septin-3 in synaptic/neuronal autophagy. (1) Septin-3 acting as an adapter between autophagophore-bound LC3B and selective presynaptic cargo escorting it for autophagic degradation. (2) Septin-3 binding to partner motor protein myosin 1B could play role in autophagosome-positioning, or autolysosome biogenesis, since MYO1B is located on lysosomes. (3) TRκB is located on late endosomes and signaling endosomes. Septin-3 is reported binding TRκB. Septin-3 therefore could play a role in autophagosome‒late endosome interaction. (4) CDC10, orthologue of septin-3 in yeast localizes to endosomes and autophagosomes and is assumed to play role in membrane-transport to autophagosome. This assumption stands for septin-3 in mammalian neurons. **B** Possible connections of septin-3 accumulation, neuro-immunological pruning and autophagy

## Discussion

In this study, we provide evidence that septin-3 has a functional LIR motif and binds to Atg8 homologs LC3B and GABARAPL2 with comparable, high nanomolar affinity. Moreover, we show that due to autophagy induction with AUTEN-67, colocalization of septin-3 with LC3B increases in primary neurocortical cell cultures. Septin-3 levels change according to the levels of selective autophagy receptor p62 and membrane associated autophagosome marker LC3B-II. Besides finding septin-3 on LC3B positive structures, we also found several septin-3 positive mitochondria. We examined if colocalization of septin-3 to the early mitophagy marker PINK1 can be enhanced with mitophagy induction using CCCP, but found no correlation, however, LC3B and septin-3 colocalization increased in these samples, as well. We conclude that septin-3’s function can be connected to neuronal autophagy.

Importantly, certain septins have already been connected to autophagy in non-mammalian cells. Fungal septin-1 and 3 (spn1 and spn3, not orthologs of mammalian septins) form a filamentous bundle in response to glucose starvation, a well-known autophagy-inducing method [72]. An additional relevant work that probably strengthens our conclusions is the research of Barve and colleagues [73, 74]. They examined septins in yeast (*Saccharomyces cerevisiae*) and found, if septins CDC10, CDC11 and SHS1 were mutated, pexophagy (autophagy of peroxysomes) and general autophagy recoiled. Septins formed a ring at pre-autophagic structure, while CDC10 and SHS1 localized further to autophagosomes. They hypothesized that these septins get to the pre-autophagic structure by the endocytic pathway [73, 74]. It is important to mention that based on the phylogenetic analysis of septins, the above-mentioned CDC10 and mammalian septin-3 are orthologues with a close relation [75], and therefore could act similarly in mammalian cells.

In mammals, septin-6 and 7 have been connected to autophagy in pathogen defense [76]. A recent publication showed that reducing septin-5 levels increased autophagy of amyloid-precursor protein (APP) and septin-5 localized to LC3B positive autophagosomes, proposing septin-5 as a negative regulator of autophagy [77]. In accordance with this, in our previous work, we have also detected septin-5 accumulation in C1q-tagged synapses to a higher extent in APP/PS1 animals compared to wild-type ones, although, without correlation between the quantity of septin-5 and the amount of synaptically bound C1q [30]. This raises the question if septin-3 and septin-5 works contrarily in autophagy.

Considering our results, elevation in septin-3 levels might indicate ongoing autophagy or disturbances in the late phases of autophagy. By connecting this hypothesis to our previous results [30], where septin-3 is accumulated in degradable synapses proportionately to surface-bound C1q, autophagy could be linked to complement-mediated synaptic pruning. This concept can be supported by previous reports from the literature. Synaptic pruning and autophagy have been connected earlier, when impaired autophagy caused pruning defects of dendritic spines and autism-like pruning deficits [78]. In C1q-mediated synaptic pruning, synapses show an elevation in the levels of cleaved caspase-3 [31] and mitochondrial reactive oxygen species (ROS) accumulation [30]. Light-activated ROS production induced presynaptic autophagy in mouse hippocampal neurons [79]. Concerning elevated caspase-3, Yang and colleagues found elevated caspase-3 accumulation in autophagic vesicles of APP/PS1 mice neurons after inhibiting its degradation with cysteine protease inhibitor [80]. Most importantly, extracellular C1q treatment induced elevation in the expression of cleaved caspase-3 and the amount of membrane-associated LC3 and autophagosomes, simultaneously, in bronchial epithelial cells, leading to cell death with autophagy [81]. Similar mechanisms might occur in C1q-tagged synapses that are prone to pruning.

Based on our results and the literature, we could speculate the following roles for septin-3 in autophagy:

(i) The synaptic septin-3 has the characteristics of an *autophagy receptor*, having a functional LIR motif and binding LC3B with a moderate affinity. During autophagy induction in cortical neurons, septin-3 can be found on double-membraned and LC3B positive structures. Moreover, septin-3 levels changes similarly to the selective autophagy receptor p62 during autophagy induction, early and late autophagy inhibition, in neurons. By binding LC3B and a synaptic cargo (such as degradable proteins, cytoskeletal structures or organelles) simultaneously, that septin-3 could escort to the forming phagophore solitarily, or by binding adaptor proteins (Fig. 8A1). We found mitochondrion-bound septin-3 in cortical slices (Fig. 7A), and examined the possibility of septin-3 binding to PINK1-tagged dysfunctional mitochondria as cargo, but found no change in colocalization between septin-3 and PINK1 after mitophagy-induction (Fig. 5E). We conclude that septin-3 likely does not act as a specific mitophagy receptor.

(ii) Nevertheless, LIR motifs can be found in autophagy-related, but non autophagy-receptor proteins that play role in *autophagosome transport or maturation* [33]. A recent study revealed that another septin from the SEPT3 subgroup, septin-9 acts as an adaptor protein by recruiting dynein-dynactin to lysosomes and enhances their retrograde transport during stress [82]. Septin-9 also binds a kinesin [83]. Septin-3 also has a motor protein binding partner, myosin IB (MYO1B), a non-conventional, one-headed myosin found in axons [34, 84]. MYO1B associates to tubulovesicular organelles, such as the Golgi apparatus, ER, endosomes, lysosomes, multivesicular bodies and may play role in local transport of organelles and, importantly, autophagosome-lysosome fusion [84, 85]. We also detected septin-3 at microtubules on electron micrographs (Fig. S3B). Thus, septin-3 could help in the positioning of the autophagosome, or in the fusions of the autophagic membrane (Fig. 8A2).

(iii) CDC10, the orthologue of septin-3 co-localizes with endosome markers in yeast [74]. It also has been reported that TrκB binds to septin-3 [86]. TrκB, a BDNF receptor localize to signaling endosomes, late endosomes and amphisomes (fusion of late endosomes and autophagosomes). Fusion of activated TrκB-containing late endosomes with autophagosomes guarantees the long-distance transport of activated TrκB to the soma on the outer membrane of the amphisome, and contributes to BDNF-dependent gene transcription [87]. Septin-3 could participate in the interplay of TrκB-amphisome biogenesis (Fig. 8A3) or membrane transport from recycling endosomes or tubulovesicular organelles to the phagophore (Fig. 6A4).

Neuronal autophagy is crucial to maintain proper synaptic functioning. Autophagosomes form in the distant axonal compartments (but, to the best of our knowledge, not in dendrites) and travel to the cell body to be degraded [87]. This mechanism modulates neuronal transmission by reducing the size of ER by ER-phagy in presynapses [88], contributes to synaptic plasticity in many other ways [89], and plays a protective role during various stress conditions [90]. Due to autophagic failure, autophagosome accumulation in synapses characterizes AD [91, 92] and this accumulation leads to increased Aβ production [93]. In light of this, overflowing changes in presynaptic septin-3 might alter neuronal autophagy and worsen the chances of successful defense against AD. However, knock out experiments of septin-3 and septin-5 (latter is recently discovered as negative autophagy regulator [77]) resulted changes in other septins’ levels in murine brain [25], and therefore redundancy of septin-3 function in the neuronal autophagic system must be examined.

In summary, we introduced LC3B as a new binding partner of septin-3, proving their direct interaction. We provided binding affinities of septin-3 with Atg8 homologues LC3B and GABARAPL2. Based on sequence analysis, molecular dynamics simulations and fluorescence spectroscopy we proposed the interacting septin-3 LIR motif. Septin-3’s main function has not yet been described. Our results raised the possible involvement of septin-3 in neuronal autophagy. We have proven that septin-3 levels change accordingly to autophagy proteins during autophagy modulation, and that colocalization with LC3B increases with autophagy enhancement and blockade in primary neurons. Our results also extend the knowledge on septin-3 localization in neurons as we found septin-3-positive autophagic vesicles, MVB-like structures and mitochondria. The detailed mechanism and dominant role of septin-3 in autophagy is yet to be examined, however we proposed new hypotheses of function. Our results serve as a starting point for future studies to discover how septin-3 contributes to synaptic/neuronal autophagy and put septin-3 related research (e.g., relation to AD or neuronal development) into new perspectives.

## Supplementary Information

Below is the link to the electronic supplementary material.

Supplementary file1 (PDF 409 KB)

## Data Availability

The datasets used and/or analyzed during the current study are available from the corresponding author on reasonable request.
