# Supplementary material for: Neuronal-specific septin-3 binds Atg8/LC3B, accumulates and localizes to autophagosomes during induced autophagy
Source: Cell Mol Life Sci. 2022 Aug 6;79(9):471. doi: 10.1007/s00018-022-04488-8 (PMC9356936; doi:10.1007/s00018-022-04488-8)
Supplement: Supplementary file 1 — Supplementary file1 (PDF 409 KB) [file 18_2022_4488_MOESM1_ESM.pdf]

SUPPLEMENTARY INFORMATION

Neuronal-specific septin-3 binds Atg8/LC3B, accumulates and localizes to autophagosomes during induced autophagy

Cellular and Molecular Life Sciences

Vilmos Tóth[1], Henrietta Vadászi[1], Lilla Ravasz[1], Dániel Mittli[1], Dominik Mátyás[1], Tamás Molnár[2], András Micsonai[1], Tamás Szaniszló[2], Péter Lőrincz[3], Réka Á. Kovács[1], Tünde Juhász[4], Tamás Beke-Somfai[4], Gábor Juhász[1,5], Balázs András Györffy[1,6], Katalin A. Kékesi[1,7,8], József Kardos[1,*]

[1]ELTE NAP Neuroimmunology Research Group, Department of Biochemistry, Institute of Biology, ELTE Eötvös Loránd University, Budapest, Hungary

[2]Department of Biochemistry, Institute of Biology, ELTE Eötvös Loránd University, Budapest, Hungary

[3]Department of Anatomy, Cell and Developmental Biology, Eötvös Loránd University, Budapest, Hungary

[4]Institute of Materials and Environmental Chemistry, Research Centre for Natural Sciences, Budapest, Hungary

[5]CRU Hungary Ltd., Göd, Hungary

[6]Department of Neuroscience, University of Copenhagen, Copenhagen, Denmark

[7]Department of Physiology and Neurobiology, Eötvös Loránd University, Budapest, Hungary

[8]Laboratory of Proteomics, Institute of Biology, ELTE Eötvös Loránd University, Budapest, Hungary

[*]Correspondence: kardos@elte.hu

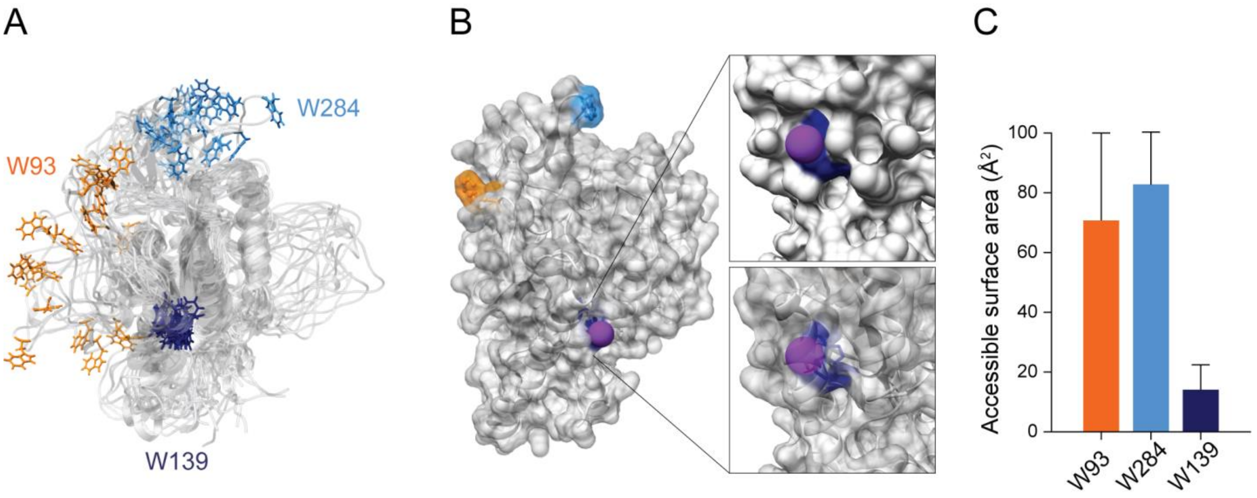

Fig. S1 Mobility and accessibility of septin-3 Trp-s. (A) Superpositional MD models representative of mobility of Trp sidechains. Core LIR4 and corresponding W284 is marked light-blue, LIR1 W139 is marked dark-blue. Non-LIR W93 is marked orange. (B) Iodide placed at LIR1 W139. (C) Iodide accessibility of septin-3 Trp sidechains.

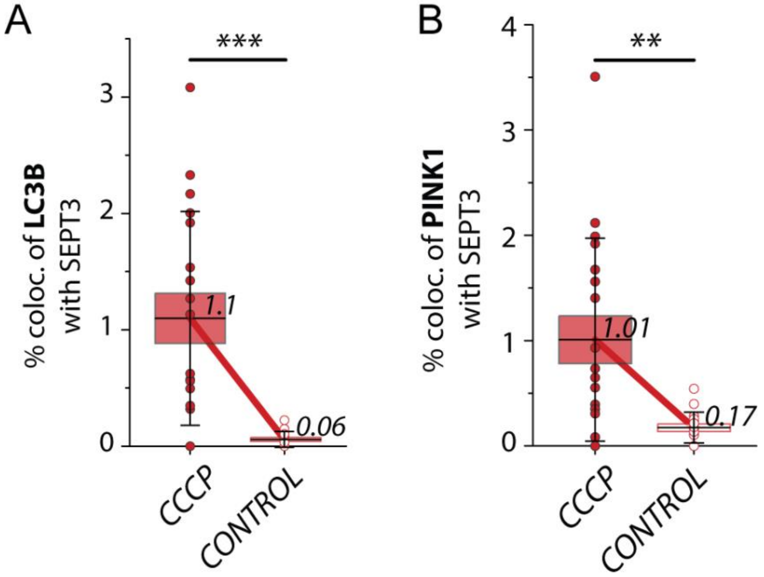

Fig. S2 Septin-3 colocalizing LC3B and PINK1 in CCCP treated primary neurons. (A) SEPT3 colocalizing LC3B. CCCP treated: 1.10% ± 0.22%; 0.92%. Controls: 0.06% ± 0.02%; 0.07%. Significance: p=5×10-5. (B) SEPT3 colocalizing PINK1. CCCP treated: 1.01% ± 0.23%; 0.96%. Controls: 0.17% ± 0.04%; 0.15%. Significance: p=0.0012. Means are marked with horizontal lines with values; boxes represent standard error of the mean (SEM). Flags indicate standard deviation (SD).

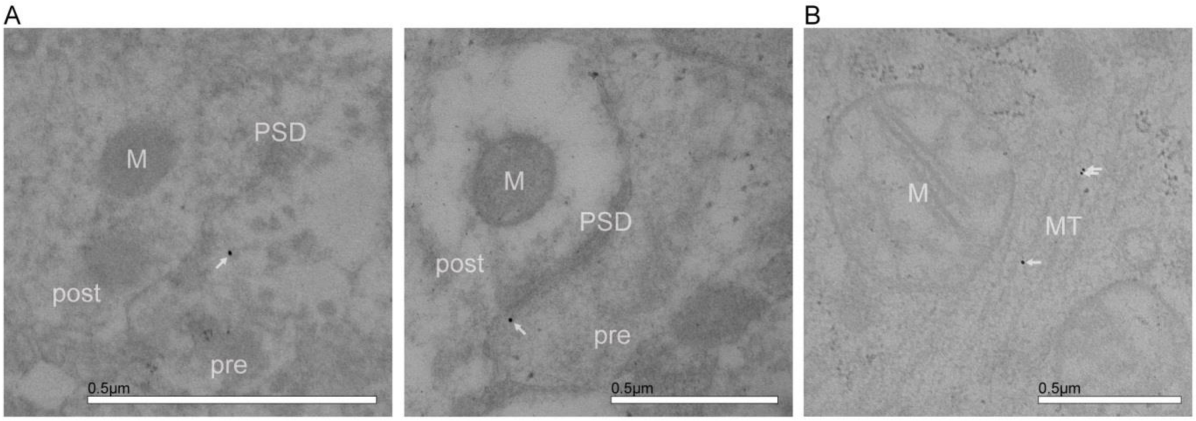

Fig. S3 Electron micrographs of mouse brain slices, with septin-3 immunogold staining. (A) Septin-3 localization at the synapse, at the presynaptic (pre) compartment. Postsynapse (post), postsynaptic density (PSD) and postsynaptic mitochondria (M) are marked. (B) Septin-3 localized on microtubules (MT) near mitochondria (M).

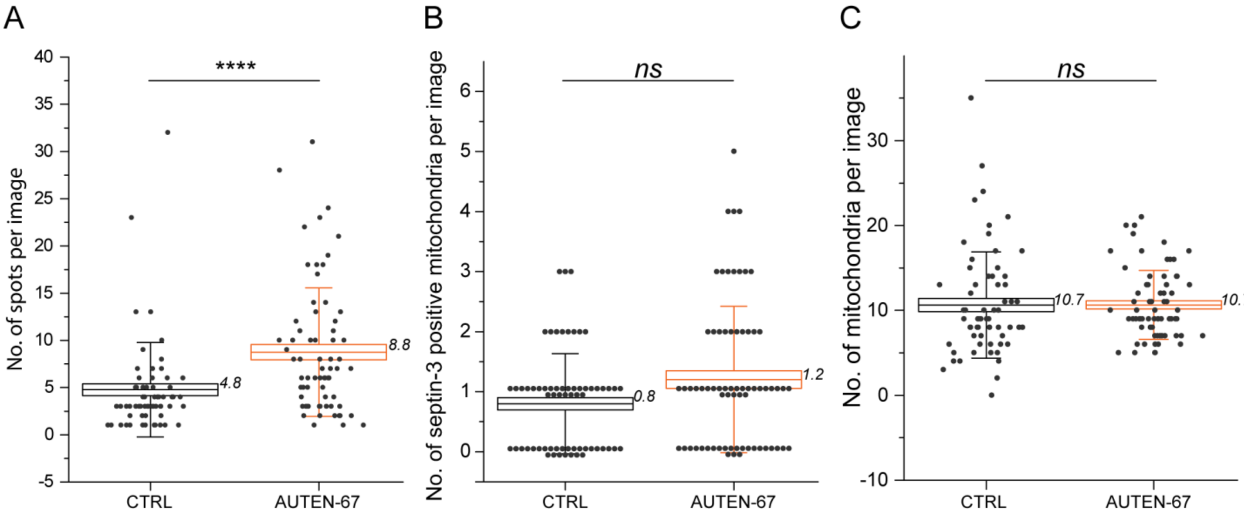

Fig S4 Quantification of TEM images. A total of 134 images were analyzed from five-five cortical cortex slices on formvar-carbon coated grids from AUTEN-67 treated and control samples. (A) Counted numbers of colloidal gold particles per image. Numbers significantly differ ($p<0.00001$) in AUTEN-67 treated samples (8.75 ± 0.82) and control samples (4.77 ± 0.62), that correlate with western blot results on septin-3 accumulation in AUTEN-67 treated cells (Figure 6A). (B) No significant difference can be found in the number of septin-3 positive mitochondria, observed per image (controls: 0.8 ± 0.1; AUTEN-67 treated: 1.2 ± 0.15) (C) No difference can be found in the average number of mitochondria observed on images (controls: 10.65 ± 0.78; AUTEN-67 treated: 10.65 ± 0.49). Data are presented on box diagrams representing mean with horizontal lines (also shown with value). Boxes represent standard error of the mean (SEM), flags indicate standard deviation (SD). Mann-Whitney U test statistics were used to determine significance. Data in the text presented as mean ± SEM.
